# Companion dog in nursing home: A study on mechanisms of efficacy and implementation

**DOI:** 10.1093/geroni/igaf122.399

**Published:** 2025-12-31

**Authors:** Bobo Hi Po Lau, David Cheung, Stephanie Law, Miu Yee Lee, Luis Miguel Dos Santos, Joe Tsz Kin Ngai, Sandrine Man-chi Chung

**Affiliations:** Hong Kong Shue Yan University, North Point, China (People’s Republic); Hong Kong Seeing Eye Dogs Services, Ta Kwu Ling, Hong Kong, China (People’s Republic); Cultural Homes, Tsuen Wan, Hong Kong, China (People’s Republic); Caritas Li Ka Shing Care & Attention Home, Tuen Mun, Hong Kong, China (People’s Republic); Hong Kong Shue Yan University, North Point, Hong Kong, China (People’s Republic); Hong Kong Shue Yan University, North Point, Hong Kong, China (People’s Republic); Hong Kong Shue Yan University, North Point, Hong Kong, China (People’s Republic)

## Abstract

Research has shown that human-animal interactions help older adults cope with emotional difficulties and foster social interactions. However, seldom have studies elaborated how a resident dog is engaged in a nursing home to contribute to the well-being of the residents, as well as the contextual factors that lead to practices which benefit both humans and animals. This study employed a multi-stake holder, qualitative methodology to investigate the efficacy and implementation of a ‘Companion dog’ program in two nursing homes in Hong Kong. Four focus groups were held with the staff and ten individual interviews were conducted with the residents. Our analysis revealed that human-dog bonding developed spontaneously during bed-side visits and group activities, and caused better mood, higher morale, fewer disruptive behaviors, and more social interactions among the residents. Some residents showed initiative and even engaged in taking care of the dogs. While the resident dogs did not significantly increase staff workload, adequate manpower, a large premises with outdoor recreational area, commitment of the staff to animal welfare, and receptiveness of the families were all vital for the success of the program. Additionally, close collaboration with the dog trainer is pivotal for ensuring the well-being of the dogs. Despite the success of the four-month program, post-program evaluation revealed the need for the resident dogs to break before continuing their tenure. A combination of a resident dog program with intermittent visits from other therapy dogs may enhance animal well-being and the therapeutic effectiveness of the dog-assisted activities.

